# Gastrointestinal stromal tumors: correlation between symptoms at presentation, tumor location and prognostic factors in 47 consecutive patients

**DOI:** 10.1186/1477-7819-9-13

**Published:** 2011-02-01

**Authors:** Salvatore Caterino, Laura Lorenzon, Niccolò Petrucciani, Elsa Iannicelli, Emanuela Pilozzi, Adriana Romiti, Marco Cavallini, Vincenzo Ziparo

**Affiliations:** 1 Surgical and Medical Department of Clinical Sciences, Biomedical Technologies and Translational Medicine, Faculty of Medicine and Psychology University of Rome "La Sapienza" Italy; 2 Department of Radiology, Sant'Andrea Hospital, Faculty of Medicine and Psychology University of Rome "La Sapienza, Italy; 3 Department of Pathology, Sant'Andrea Hospital, Faculty of Medicine and Psychology University of Rome "La Sapienza", Italy; 4 Department of Oncology, Sant'Andrea Hospital, Faculty of Medicine and Psychology University of Rome "La Sapienza", Italy

## Abstract

**Background:**

Gastrointestinal stromal tumors (GIST) are mesenchymal tumors of the gastrointestinal tract, usually kit-positive, that are believed to originate from interstitial cell of Cajal, or their related stem cells. The most common clinical presentation of these tumors is gastrointestinal bleeding, otherwise they may cause intestinal obstruction, abdominal pain, a palpable mass, or can be incidentally detected during surgery or endoscopic/radiological procedures. Prognosis is related to the size of the tumor and to the mitotic rate; other prognostic factors are tumor location, tumor resection margins, tumor rupture, and c-kit mutation that may interfere with molecular target therapy efficacy.

**Aim:**

Primary aim of this study was to report our experience regarding GIST patients, correlating symptoms at presentation with tumor localization and risk factors.

**Patients and methods:**

47 consecutive patients undergone to surgical resection for GISTs were enrolled in a prospective study from December 1999 to March 2009. Patient's clinical and pathological features were collected and analysed.

**Results:**

The most common symptom was abdominal pain. Bleeding in the digestive tract and abdominal pain were more frequent in gastric GISTs (58% and 61%); acute abdominal symptoms were more frequent in jejunal and ileal GISTs (40% and 60%), p < 0.05. We reported a mild correlation between the mitotic rate index and symptoms at presentation (p 0.074): this correlation was stronger if GISTs causing "acute abdominal symptoms" were compared with GISTs causing "abdominal pain" as main symptom (p 0.039) and with "incidental" GISTs (p 0.022).

We observed an higher prevalence of symptomatic patients in the "high risk/malignant group" of both the Fletcher's and Miettines's classification (p < 0.05).

**Conclusion:**

According with our findings symptoms correlate to tumor location, to class risk criteria as mitotic index and risk classifications, however we cannot conclude that symptoms are *per se *predictive of survival or patient's outcome.

## Background

Gastrointestinal stromal tumors (GIST) are mesenchymal tumors of the gastrointestinal (GI) tract, usually kit-positive, that are believed to originate from interstitial cell of Cajal (ICC), the gut pacemaker of the autonomic nervous gut system, or their related stem cells [1,2]. GISTs usually occur in adults, with a median age of 55-60 years and incidence of 10 to 20 new cases per million/year [3]. GISTs represent 80% of mesenchymal gastrointestinal tumors and 0.1-3% of all gastrointestinal malignancies [4-7]. GIST's pathogenesis is related to kit and platelet-derived growth factor receptor alpha (PDGFR alpha) mutation. kit and PDGFR alpha encode for similar type III receptor tyrosine kinase proteins: these mutations are somatic and occur only in the neoplastic tissue of sporadic GISTs, whereas constitutional mutations in familial GISTs occur in every cells of the body and are inheritable [8-11].

GISTs have specific immunohistochemical (IHC) markers: 95% are CD-117 positive, 70-80% are CD34 positive, and 20-30% are smooth muscle actin (SMA) positive, whereas desmin is positive in less than 5% of GISTs [1,12]. Discovered on GIST (DOG) 1, known also as ANO1, has emerged in recent years as a promising biomarker for GISTs, since recent studies documented that DOG1 antibodies are more sensitive than kit antibodies in detecting gastric GISTs and tumors carrying PDGFR alpha mutations [13].

GISTs may develop through all the GI tract: 50-70% in the stomach, 25-30% in the small intestine, 5-10% in the colon-rectum, < 5% in the esophagus, the remaining may arise within the omentum or within the peritonel layers (Extra-Gastro-intestinal Stromal Tumors, EGISTs) [12,14,15]. Familial GISTs are very rare, occurring in patients with inheritable germline kit or PDGFR alpha mutations; 5% of GISTs occur in patients with Neurofibromatosis type 1 syndrome and in Carnery triad (gastric GIST, paraganglioma, pulmonary chondroma) [1].

The most common clinical presentation of these tumors is GI bleeding (with acute hematemesis, melaena, or chronic anemia); they may cause GI obstruction, abdominal pain, weight loss or a palpable mass, otherwise they can be incidentally detected during surgery or endoscopic/radiological procedures [6,16-18].

Prognosis is related to the size of the tumor and to the mitotic rate: tumors > 10 cm or with a mitotic rate of >5 per 50 HPF have a higher risk of recurrence and metastatic spread and are associated with a poor prognosis (approximately 20-30% of GISTs). Other prognostic factors are tumor location, the persistence of tumor residuals within the surgical resection margins, tumor rupture, and c-kit mutation that may interfere with molecular target therapy efficacy [19-22].

State of the art in GIST's treatment is based on two gold standards: surgery and target molecular therapy.

The aim of surgical treatment is complete resection, avoiding tumor rupture, preferring wedge resections whenever possible; lymphadenectomy is not recommended due to the rarity of nodal metastasis, with the exception of GISTs occurring in a setting of Carney triad, that usually show an higher rate of lymph node metastasis [23].

The majority of kit-mutant proteins are sensitive to agents that block KIT and PDGFR alpha, like Imatinib, with a response rate that reaches almost 70% even in advance disease [24], however resistance to the therapy has been widely reported [25].

The aim of the present study was to report our experience on 47 consecutive patients undergone to surgical resection for GIST tumors, correlating symptoms at presentation with a) tumor's localization, and b) risk factors and classification.

## Patients and Methods

### Study protocol and patients

Fifty-one consecutive patients referred for surgical treatment for GIST tumors were enrolled in a prospective-observational study: thirteen patients (from December 1999 to March 2003) at the Department of Surgery "Pietro Valdoni" of University of Rome "La Sapienza", and thirty-eight patients (from April 2003 to March 2009), were recorded, by the same surgical team, at Sant'Andrea Hospital of Rome, Faculty of Medicine and Psychology, University of Rome "La Sapienza".

Within this group, only patients presenting with a primary GIST tumors, surgically resectable, were selected. Patients presenting with metastatic disease, were first referred to the Oncology Unit before surgical treatment, and then selected if surgical resection was indicated as primary treatment. Three patients not suitable for surgical treatment due to co-morbidity, and due to spreading of the disease were excluded; one patient was excluded because presenting with a recurrence of a GIST previously treated else-where.

According with these criteria, forty-seven patients out of fifty-one observed, were enrolled in an observational study. Authorization of the ethical board was not required for this study, but signed consent for treatment and evaluation of the data was obtained from all selected patients.

Patients were staged through chest and abdominal computer tomography (CT) scan; magnetic resonance (MR), was employed as a second-line imaging assessment, at radiologist's discretion. Endoscopic ultrasound scan (EUS) was employed to support diagnosis and define mucosal infiltration of four gastric GISTs. Rectal GISTs were staged through trans-anal ultrasound and MR.

The aim of surgical treatment was a complete surgical resection with negative resection margins (R0), sparing the organ whenever possible, without lymphadenectomy. Laparoscopic resection was performed whenever possible, according with tumor size (2-5 cm) and tumor localization.

R1 (persistence of microscopic tumor deposits within the resection margins) and R2 (persistence of macroscopic tumor residual) resections, or relapse of disease after R0 resection, presence of high risk factors criteria or metastases at the time of surgical procedure were considered as indications for adjuvant therapy. Histological processing was obtained with formalin fixation followed by paraffin inclusion, 3 μm sectioning, and staining with hematoxylin and eosin. Morphologic appearance and cellular descriptions referred to standard descriptions as epithelioid, spindle and mixed cells. Mitotic Index (MI) was obtained counting mitotic features in 50 consecutive microscopic high-powered fields (HPF), 400×. IHC processing was carried through antibodies for CD117 (c-kit), S-100 protein and CD-34 and SMA-alpha. Patients showing recurrence/persistence of the disease at follow-up were investigated for c-kit mutation profile.

All patients were clinically evaluated each 3 months for the first year, each 6 months later after. Follow-up imaging evaluation was carried through PET scan, abdominal ultrasound, CT or MR scan according with the RECIST and Choi's criteria for response assessment [26].

### Classification

GISTs were classified according to risk prognostic classifications of Fletcher (NIH 2002) and Miettinen (AFIP 2006, NCCN 2007) [27,28].

### Data collection and statistical analysis

Symptoms were recorded on the admission and collected by the team. Data regarding symptoms at presentation, tumor localization, and risk factors of all selected patients were collected in a database (Visual Fox Pro 7.0, Microsoft Corporation^®^), up-dating follow-up each 3-6 months regarding symptoms, adjuvant therapy, relapse of the disease; statistical analysis was carried through the SPSS software (SPSS for Window 9.0^®^), using the t-Student's test, χ^2^'s test or Fisher's test, and Kruskal-Wallis's test; p < 0.05 was considered as statistical significance value; Kaplan-Meier survival curves and Logrank test for comparison of survival curves were obtained through the MedCalc software version 11.4.4.0.

## Results

Forty-seven consecutive patients were selected, twenty-nine males and eighteen females (M/F = 1,61), mean age 61.4 years (range: 27-84 years, median 62, DS = +-15.61); no difference of age at diagnosis was recorded between the two sex.

**Figure 1:**
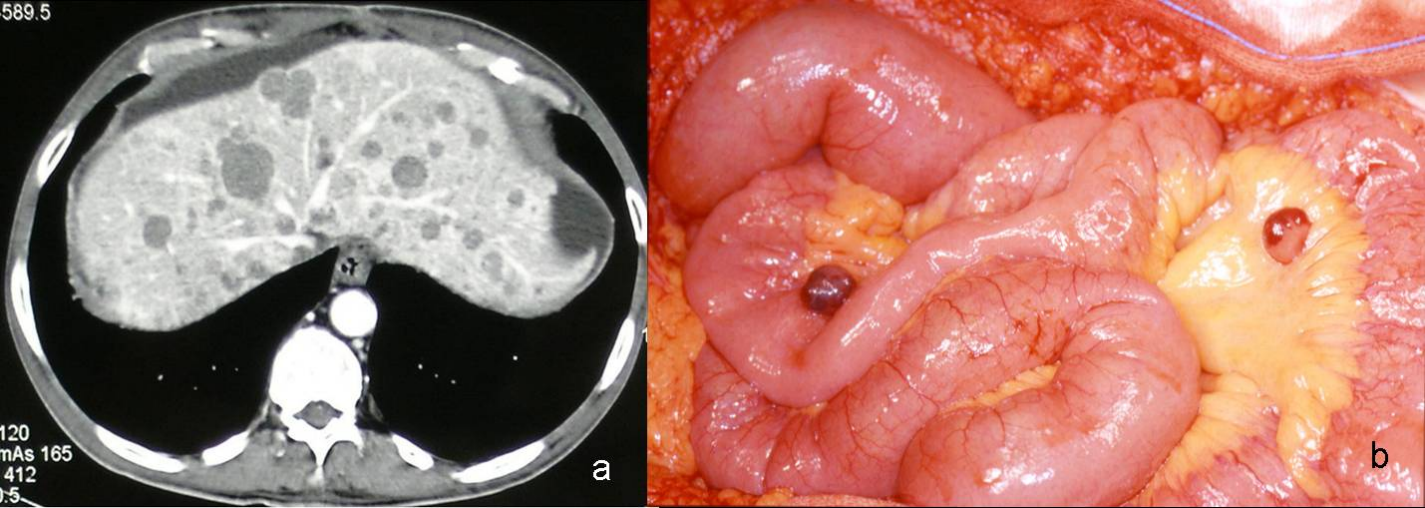
**Metastases from primary GIST tumors**. a) CT scan of liver mestastases from GIST tumor. b) Peritoneal metastases from primary GIST tumor.

Table 1 summarize tumor's localization. Twenty-eight patients had gastric GIST, three duodenal, six jejunal, five had a GIST localized within the small bowel, three rectal (one localized at the anal canal level), one had an esophageal GIST, the remaining one was considered as an EGIST, since it was localized within the mesocolon root. One patient with jejunal GIST had Neurofibromatosis type I. Seven patients (15%) had metastases at the time of diagnosis (three liver, four peritoneal; Figure 1).

**Table 1:** GIST locations and frequencies in our population study group.

Localization	Patients (n)	(%)
Gastric	28	59,6

Duodenal	3	6,4

Jejunal	6	12,8

Small bowel	5	10,6

Rectal	3	6,4

Esophageal	1	2,1

Mesocolon root (EGIST)	1	2,1

Tot	47	100%

All selected patients underwent to surgical resection: 85% elective, 15% in emergency. R0 resection was achieved in all patients, with the exclusion of one bulky GIST of the rectum, who had an R1 low anterior resection, but was successively R0 after a Miles abomino-perineal amputation. Total laparoscopic resection was achieved in seven patients, all with gastric GISTs, with a diameter range between 2 and 4.5 cm. Two patients with gastric GISTs were converted to open surgery due to the tumor's localization causing a poor visualization of anatomy and thus difficult resections.

Histology was consistent with spindle-shaped cells in 48.6%, epithelioid in 32.5%, mixed in 18.9% of the cases. Gastric GISTs showed a mild prevalence of spindle-shaped cells if compared to epithelioid and mixed pattern (50% vs 32% and 18%, p 0.056). All GISTs expressed CD117 ranging from 60% to 100%; CD34 was positive in 81%. Eighteen patients were actin positive and fifteen patients were S100 positive. IHC positivity to CD117, CD34 and S100 was not related to GIST location (p 0.096).

No peri-operative mortality was recorded; mean hospitalization was 6.9 days (range: 3-15 days). Post-operative course after laparoscopy was mildly shorter if compared with standard open surgical resections (mean 5.2 days vs 6.9 days).

### Symptoms at presentation

Table 2 summarizes the clinical presentations and tumor locations. The most common symptom was abdominal pain, mainly epigastric or periumbilical (18 patients).

**Table 2:** Clinical presentation, symptoms and GIST localization reported in our population study group.

	Clinical presentation and Symptoms				
	
GIST localization	GI bleeding	Abdominal pain	Acute abdomen	Palpable mass	Asymptomatic
Gastric	8	11	-	2	6

Duodenal	2	1	-	-	-

Jejunal	1	1	2	1	2

Small bowel	2	1	3	1	1

Rectal	1	2	-	-	-

Esophageal	-	1	-	-	-

EGIST	-	1	-	1	-

(NB. each GIST might have more then one symptom reported, eg 1 EGIST padient: abdominal pain plus papable mass, thus total ≠ 47)

Five patients had acute abdominal symptoms; associate symptoms were: vomiting, asthenia, dyspepsia, fever, weight loss and dysphagia. A palpable abdominal mass was detected in five patients (10.6%). Nine patients showed no signs or symptoms, had an incidental diagnosis and were considered asymptomatic (19.1%).

Forty patients (85%) underwent to elective resection, otherwise seven patients (15%) underwent to emergency resection: three due to bowel obstruction, three due to bleeding, one patient due to a suspected appendicitis. Four patients (1 jejunal, 2 gastric, 1 ileal) had an incidental diagnosis of GIST at laparotomy for other reasons (three colon cancers and one hepatic abscess).

Four patients had an endoscopic pre-operative diagnosis of GIST, due to a suspected gastritis, epigastralgia or to progressive weight loss.

Fourteen patients (29,8%) reported acute anemia (mean Hb 7.6 g/dl), due to high digestive bleeding in twelve patients, rectorrhagia in one patient, and enterorrhagia with hemoperitoneum in the remaining one; four patients reported a chronic sideropenic anemia.

### Tumor location

**Figure 2:**
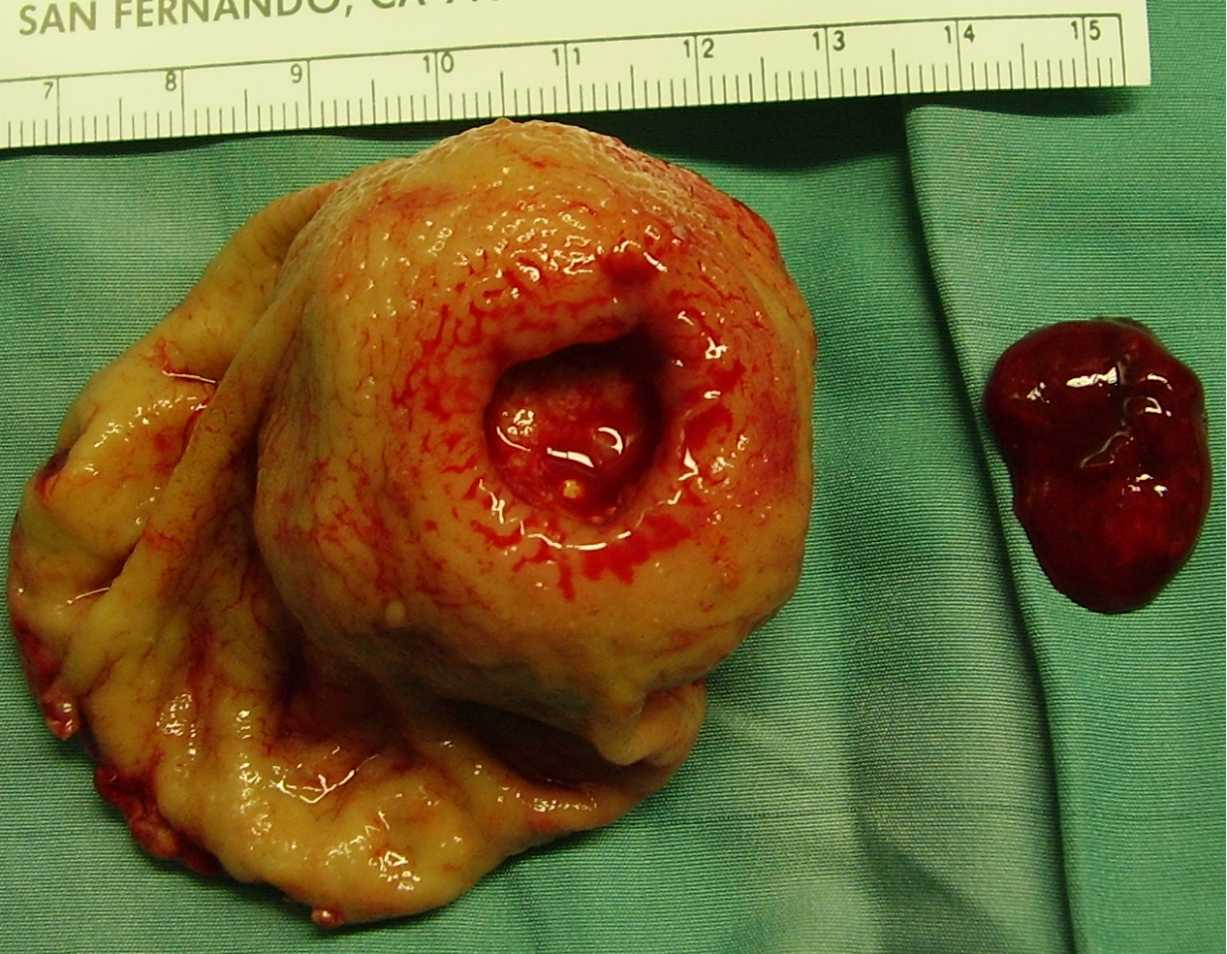
**Surgical specimen of a jejunal GIST: the blood clot placed on the right side revealed the tumor causing the mucosal ulceration**.

Bleeding was mainly reported in gastric GISTs, however this was due to the higher rate of gastric GIST in our population (Table 2). On the basis of this background, patients reporting mucosal ulceration and bleeding, were stratified according with tumor's localization: results of this analysis documented that these symptoms were seen more frequently in duodenal GISTs (2 out of 3 patients, 66%), rectal GISTs (1 out of 3 patients, 33%), comparing with gastric GISTs (8 out of 28 patients, 28%) and jejunal GISTs (1 out of 6 patients, 16%) (Figure 2).

Statistical analysis showed that bleeding in the digestive tract and abdominal pain were more frequent in gastric GISTs comparing with other localizations (58% and 61%) otherwise acute abdominal symptoms were more frequent in jejunal and ileal GISTs (40% and 60%), p < 0.05.

Indeed in our experience, five out of seven patients undergone to an emergency surgical resection (three due to a bowel obstruction and two due to GI bleeding), had jejunal/ileal GISTs.

### Risk factors and classification

Mean maximum tumor diameter (MMTD) was 7,4 cm (median 5.0, DS +- 6,72, range 1-33 cm). Tumor size was not related to age, sex or location (gastric vs other locations p 0.808).

Gastric GISTs showing abdominal pain were larger in size than those showing bleeding as main symptom, but statistical analysis failed in detecting a significant difference within these groups (p 0.077); similar jejunal/ileal GISTs showing acute abdominal symptoms were larger in size (with a mean diameter of 13.2 cm) comparing with jejunal/ileal GISTs showing other symptoms at presentation, the difference, however did not reach a significant value (p n.s.).

Thirty-five patients had MI <5/50 HPF (74.5%), otherwise four patients (8.5%) ranged between 5 and 10/50 HPF and eight patients (17%) had a MI > 10/50 HPF.

Results of our analysis showed a correlation between MI and tumor's location, since there was a significant difference if gastric GISTs were compared with duodenal GISTs (p 0.018).

We reported a mild correlation between MI and symptoms at presentation (Kruskal-Wallis test H = 7.532, 3 gl, p 0.074); this correlation was stronger if GISTs causing "acute abdominal symptoms" were compared with GISTs causing "abdominal pain" as main symptom (p 0.039) and with "incidental" GISTs (p 0.022).

Patients were stratified according with Fletcher's and Miettinen's risk factor criteria: Table 3 shows differences within these two classifications and the consequent re-distribution of patients in our population study group regarding tumor localization and risk factors groups. However, even due to the small number of investigated patients, we did not observe significant differences in the outcome of patients who migrated in another *status*. Table 3 shows also differences in tumor's diameter of GISTs, the surgical procedures and prevalence of symptoms in our population study group. We observed an higher prevalence of symptomatic patients in the "high risk/malignant group" of both the Fletcher's and Miettines's classification (Chi square p 0.03 and p 0.04 respectively).

**Table 3:** Fletchers's and Miettinen's Classification of GIST tumors: distribution of symptoms, tumor locations, tumor's diameter and surgical procedures in our population study group.

	Fletcher's Classification (NIH 2002)					Miettinen's Classification (AFIP 2006, NCCN 2007)			
	
	Very Low	Low	Intermediate	High	P	Benign	Intermediate	Malignant	P
**Symptoms**									
Asymptomatic	2	6	1	0	0.03	6	3	0	0.04
Symptoms	2	12	9	15		13	7	18	

**Localizations**									
Gastric	2	13	7	6		17	5	6	
Duodenal	0	3	0	0		0	2	1	
Jejunal	1	1	0	4		1	1	4	
Small bowel	0	1	2	2		0	1	4	
Rectal	1	1	0	1		1	1	1	
Other localization	0	0	1	1		0	0	2	

**Maximum diameter**									
(Mean cm)	2.0	3.5	6.0	15.3	0.01	3.48	4.35	13.33	0.01

**Surgical procedures**									
Elective resection	4	16	9	11	NS	17	9	14	NS
Emercency resection	0	3	1	3	NS	2	1	4	NS

NS: not significant, Statistical analysis: Chi-square test.

### Follow-up and outcome

**Figure 3:**
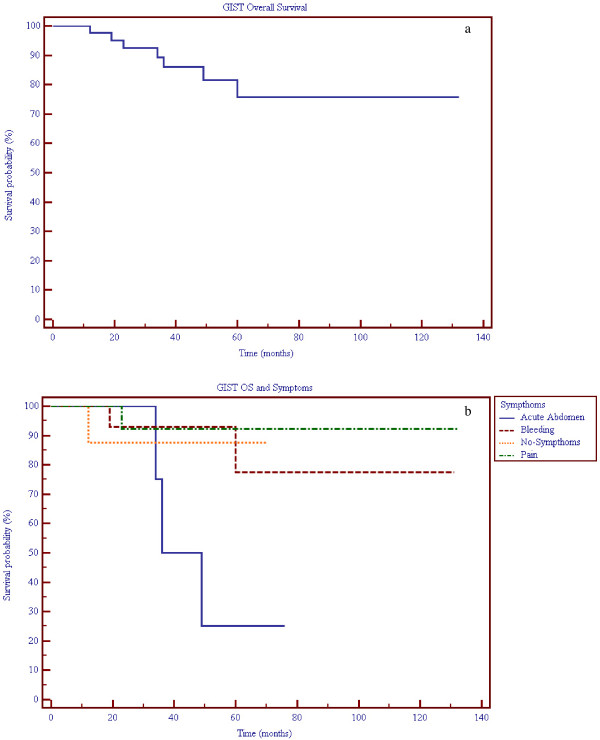
**Kaplan-Meier survival curves for GIST patients**. a) Overall Survival (OS) of GIST patients, sample size: 42. b) OS of GIST patients regarding symptoms at presentation, sample size 41: Acute abdomen 4 patients, Bleeding 14 patients, Pain 14 patients, No symptoms 9 patients (Logrank test, significance P = 0.0471).

Last update of follow up has been conducted on December 2010. Mean follow up was 54 months (median 40.5, range 6-132 months). Five patients were lost to follow-up. Figure 3a shows overall survival (OS) in our population study group. Seven patients died in the follow-up period (range of time to death 6-55 months): five patients died for other reasons (colorectal cancer, lung cancer, leukemia, Alzheimer disease, myocardial stroke), and among these three were gastric GISTs, one ileal and one rectal; two patients died because of disease progression (one jejunal, one ileal) but had yet metastases at the time of surgical resection.

It seems important to highlight that all deaths occurring in the gastric group were related to other diseases, and none of them were related to the disease progression.

Figure 3b shows the comparison of the overall survival curves regarding symptoms: patients presenting with an acute abdomen had a worse outcome comparing with patients presenting with other symptoms (Long rank test p 0.047). It is important to highlight, however, that the two out of three patients who died within this group were those two ones who died for disease progression but had yet metastases at the time of the surgical resection, thus the "acute abdomen" symptom cannot be considered *per se *predictive of OS.

Nine patients are still under pharmacological treatment: five patients with no sign of disease progression (one gastric, two jejunal, one ileal and one rectal GIST), four showing disease progression (two jejunal, one gastric, one EGIST), one of those requiring a second line therapy.

## Discussion

GISTs originate from ICC or from mesenchymal stem cells throughout the gastrointestinal tract, or in extra-gastrointestinal structures. EGISTs might present with different and not specific symptoms and usually have an aggressive behaviour. GISTs usually affect males and females with the same rate, however, we reported a mild prevalence of males, with a M:F rate of 1.61. According with published literature we reported a peack of incidence at the 6^th ^decade.

An association between GISTs and other synchronous tumors, mainly gastrointestinal adenocarcinomas, has been previously reported [29], even if this association could be incidental. We reported four GISTs presenting associated with colorectal cancers (8.5% of our series) and one patient who presented a synchronous GIST and lung cancer (2.1% of our series).

Pre-operative histological diagnosis is very uncommon: Horowitz [30] reported 50% of success rate, higher if obtained with ultrasound endoscopy; however percutaneous biopsy is not recommended due to the high dissemination risk.

Clinical presentation is usually related to tumor location, biological features, and disease spread.

GISTs might be asymptomatic in 4-53% and diagnosis might be incidental: indeed small tumors may be incidentally detected during radiologic, endoscopic or surgical procedures. De Matteo reported a mean of symptoms duration of 6 months before diagnosis [31]. According with our results, 19.2% of patients had an incidental diagnosis of GIST.

Most common symptoms are abdominal, mainly epigastric pain (usually a late symptom due compression of nearby organs) and gastrointestinal bleeding: this was reported from 17-53% of the cases, usually due to submucosal tumors causing compression, ischaemia or infiltration of the up-leading mucosa, and therefore bleeding [32-40].

The most common symptom in our experience was abdominal pain (38%), followed by bleeding in the digestive tract (29.8%).

Dysphagia characterized esophageal GIST, tenesmus rectal GIST. Asthenia is due to anemization. Palpable abdominal mass is usually reported in larger gastric or jejunal tumors [32-40]: we reported five patients (10.6%) with palpable tumors, all with a diameter > 10 cm.

GISTs causing mucosal ulceration and bleeding usually have a larger diameter, however we also reported two gastric GISTs with diameter < 3.5 cm, and two duodenal GISTs with diameter < 2 cm causing digestive bleeding. Acute abdominal symptoms are reported in 3-17% of the cases [33-41]. In our experience, there was a higher prevalence of acute symptoms in the jejunal/ileal GISTs: indeed five patients out of seven undergone to an emergency surgical resection (three due to bowel obstruction and two due GI bleeding), had jejunal/ileal GISTs.

A higher prevalence of symptomatic patients was seen in the "high risk/malignant group" of both the Fletcher's and Miettinen's Classifications (Table 3, p < 0.05).

We observed a higher prevalence of high MI in patients presenting with an acute abdomen comparing with patients presenting with pain or incidental GISTs, and moreover patients presenting with acute abdomen reported a worse survival curve if compared with other presentation, however, two out three patients who died in this group had yet metastases at the time of diagnosis.

Even if symptoms cannot be considered *per se *predictive of survival and outcome, it is however likely that those ileal GISTs presenting with acute abdomen (e.g. bowel obstruction) are those tumors with an higher mitotic rate and thus an higher rate of metastatization and disease progression. Thus even if the statistical value of the association is weak, the patients presenting with "acute abdomen" were those more likely to have more than one unfavourable prognostic factor.

GIST surgical resections could be differentiated in "easier resections" or "difficult resections": "easier" are resections of small GIST (< 2 cm), in easy accessible locations, with higher chances of providing an R0 resection: these criteria matched 19 (40.4%) of our patients; "difficult" are resections of larger tumors (> 2 cm), in difficult location as duodenum, rectum, esophageal-gastric junction, or with an higher risk of R1 resection: 28 (59.6%) patients of our series matched these criteria (9 patients required radical demolishing procedures).

Locally advanced GISTs might be candidate to surgical resection after neo-adjuvant treatment with Imatinib: surgery should be considered after 6 to 12 months of therapy, or when the maximum of the response rate is achieved. Neo-adjuvant therapy might re-define indication to surgery, morbidity and mortality rate after surgical resection. The role of surgery for residual, relapse or metastatic disease after Imatinib however, is still under definition [42].

Laparoscopic resection is nowadays a possible choice: GISTs <50 mm in size can be treated successfully by laparoscopic surgery if not contraindicated by co-morbidities [43]. Indeed the National Comprehensive Cancer Network (NCCN) recently recommended that "gastric GISTs 5 cm or smaller may be removed through laparoscopic wedge resection" when the risk of intraoperative tumor rupture is low. GISTs larger than 5 cm may be resected using a laparoscopic or laparoscopic assisted technique with hand port, depending on the tumor location and shape, using protective plastic bag to minimize the risk of port site recurrence [44,45]. Indeed tumor location might influence surgery quality, clinical evolution and prognosis.

## Conclusion

According with our findings symptoms correlate to tumor location, to class risk criteria as mitotic index and risk classifications. However, even due to the small number of investigated patients and the single centre experience, we cannot conclude that symptoms are *per se *predictive of survival or patient's outcome.

## Competing interests

The authors declare that they have no competing interests.

## Authors' contributions

SC, MC and VZ designed the study; NP, LL, EP, AR and EI performed data acquisition; SC, NP and LL controlled quality of data and algorithms; SC, LL, EP, EI and AR performed data analysis and interpretation; SC, LL and NP performed statistical analysis; SC, LL, NP, MC and VZ prepared the manuscript and its editing; all authors contributed in reviewing the final manuscript for its approval.
